# Exploring the Systemic Immune-Inflammation Index as a novel biomarker for nonalcoholic fatty liver disease: a systematic review and meta-analysis

**DOI:** 10.1097/MS9.0000000000004097

**Published:** 2025-10-15

**Authors:** Mohit Mirchandani, Herby Jeanty, Satabdi Sahu, Hessam Nejati, Saisree Reddy Adla Jala, Abinash Mahapatro, Shika M Jain, Amir Nasrollahizadeh, Ghazalgoo Arezoo, Seyyed Mohammad Hashemi, Ehsan Amini-Salehi

**Affiliations:** a Montefiore Medical Center Wakefield Campus, Bronx, NY, USA; b The Brooklyn Hospital Center, Brooklyn, NY, USA; c MKCG Medical College and Hospital, Berhampur, Odisha, India; d Gastrointestinal and Liver Diseases Research Center, Guilan University of Medical Sciences, Rasht, Iran; e School of Medicine, Guilan University of Medical Sciences, Rasht, Iran; f Mission Hospital, Asheville, NC, USA; g Hi-Tech Medical College and Hospital, Rourkela, Odisha, India; h Department of Internal Medicine, MVJ Medical College and Research Hospital, Bengaluru, India; i Tehran Heart Center, Cardiovascular Diseases Research Institute, Tehran University of Medical Sciences, Tehran, Iran; j Cardiovascular Research Center, Hormozgan University of Medical Sciences, Bandar Abbas, Iran

**Keywords:** biomarker, inflammation, meta-analysis, NAFLD, SII, Systemic Immune-Inflammation Index, systemic inflammation

## Abstract

**Background:**

Nonalcoholic fatty liver disease (NAFLD) represents one of the most prevalent chronic hepatic disorders globally and is intricately associated with metabolic dysregulation and persistent systemic inflammation. The Systemic Immune-Inflammation Index (SII) has emerged as a promising noninvasive indicator for various inflammation-mediated conditions, including NAFLD. This meta-analysis was undertaken to assess the association between SII and NAFLD.

**Methods:**

Observational studies identified through a systematic search of PubMed, Web of Science, Embase, and Scopus up to February 2025 that examined the association between SII and NAFLD were considered eligible for inclusion. Quantitative synthesis of the data was conducted through meta-analytic techniques employing a random-effects model to derive pooled effect estimates, thereby accounting for potential heterogeneity across studies.

**Results:**

The meta-analysis demonstrated a statistically significant association between higher SII levels and NAFLD. When SII was analyzed as a continuous variable, the pooled effect size was significant (Hedges’ *g* = 0.11, 95% CI: 0.04–0.18, *P* < 0.01), indicating elevated SII levels in individuals with NAFLD compared to controls. When evaluated categorically, individuals with high SII values had increased odds of having NAFLD (OR = 1.45, 95% CI: 1.18–1.78, *P* < 0.01). Statistical power was sufficient to support the validity of the observed associations.

**Conclusions:**

SII shows a significant association with NAFLD and may serve as a valuable, noninvasive biomarker for early detection and risk stratification. Despite promising results, the presence of heterogeneity and possible publication bias highlights the need for more standardized and longitudinal research to validate SII’s clinical utility in NAFLD.

## Introduction

Nonalcoholic fatty liver disease [NAFLD; now termed Metabolic Dysfunction-Associated Steatotic Liver Disease (MASLD) per recent multinational Delphi consensus to better reflect metabolic pathophysiology and avoid alcohol exclusion^[1]^] constitutes one of the most prevalent chronic hepatic disorders globally, affecting nearly one-third of the world’s population^[2–4]^. This condition encompasses a wide pathological spectrum, ranging from benign hepatic steatosis to metabolic dysfunction-associated steatohepatitis [formerly nonalcoholic steatohepatitis (NASH)^[1]^], which may progress to advanced fibrosis, cirrhosis, and ultimately hepatocellular carcinoma^[5–7]^. Recent epidemiological data demonstrated increasing burdens in youths and young adults projected to continue through 2035, driven by obesity, diabetes, and metabolic syndrome^[8]^. The underlying pathophysiology of NAFLD is complex and multifactorial, implicating mechanisms such as metabolic dysregulation, insulin resistance, and persistent low-grade systemic inflammation^[9,10]^. In light of its rising global burden and potential for severe hepatic and extrahepatic complications, the identification of reliable, noninvasive, and cost-effective biomarkers for early detection and prognostic evaluation has emerged as a critical clinical and research priority^[11–13]^. Existing indices, such as the FIB-4 and NAFLD Fibrosis Score, have limitations, including suboptimal sensitivity for early fibrosis, reduced specificity in obese or elderly populations, and dependence on multiple biochemical inputs that may vary by laboratory^[12,14]^.

**HIGHLIGHTS**

- Nonalcoholic fatty liver disease (NAFLD) is a widespread chronic condition linked to systemic inflammation and metabolic dysfunction.
- Higher Systemic Immune-Inflammation Index (SII) levels are significantly associated with increased NAFLD risk across multiple studies.
- SII is a promising, noninvasive biomarker for NAFLD, but further standardized and prospective studies are needed for validation.

Chronic systemic inflammation is recognized as a key pathological driver in the progression of NAFLD, and it also mediates the disease’s strong associations with metabolic syndrome and cardiovascular comorbidities^[15–17]^. Among a wide array of inflammatory biomarkers, the SII has garnered increasing attention due to its integrative reflection of both immune system activity and inflammatory status. Systemic Immune-Inflammation Index (SII) is computed using the formula: platelet count × neutrophil count/lymphocyte count, thereby capturing the dynamic interplay between pro-inflammatory and regulatory immune responses^[18,19]^. Elevated SII values have been associated with a range of pathological conditions characterized by inflammatory dysregulation, including cardiovascular diseases (higher SII linked to increased risk of major adverse cardiovascular events), malignancies (poorer prognosis in various cancers), and autoimmune disorders (poorer prognosis)^[20–24]^.

Emerging evidence suggests that elevated SII levels may be associated with NAFLD, potentially indicating a disruption in the equilibrium between pro-inflammatory and anti-inflammatory mechanisms^[25–28]^. Although preliminary findings are encouraging, previous reviews lacked comprehensive meta-analyses, used inconsistent SII cut-offs, or were limited by small sample sizes, leaving the precise diagnostic and prognostic utility of SII in the context of NAFLD insufficiently characterized^[25]^. In response to this knowledge gap, the present systematic review and meta-analysis was undertaken to critically assess the association between SII and NAFLD. Through the integration of current empirical data, this study aims to elucidate the potential clinical significance of SII in the management of NAFLD and to delineate directions for future investigative efforts.

## Methods

This meta-analysis was conducted in accordance with the Preferred Reporting Items for Systematic Reviews and Meta-Analyses (PRISMA) guidelines, ensuring methodological transparency and consistency. Additionally, all procedures were performed based on the standards outlined in the Cochrane Handbook for Systematic Reviews of Interventions. The study protocol was prospectively registered in the PROSPERO database (registration number: CRD420251003804) to promote research integrity and minimize reporting bias^[29,30]^.

### Search strategy

A comprehensive literature search was performed across the electronic databases PubMed, Web of Science, Embase, and Scopus from their inception until 8 February 2025. The search strategy incorporated a combination of Medical Subject Headings (MeSH) terms and relevant keywords related to “Systemic Immune-Inflammation Index,” “Non-Alcoholic Fatty Liver Disease,” and “NAFLD.” Boolean operators (AND, OR) were employed to refine the search and ensure the inclusion of all pertinent studies. To further enhance the search, references from retrieved articles and relevant review papers were manually screened to identify any additional studies that might have been overlooked. No language restrictions were applied to minimize the risk of omitting significant literature. The search strategy adhered to PRISMA guidelines and was independently verified by two researchers to ensure accuracy and comprehensiveness. The detailed search strategy for each database is presented in Supplemental Digital Content Table S1, available at: http://links.lww.com/MS9/B2.

### Study selection and eligibility criteria

For the study selection process and determination of eligibility criteria, all retrieved records were initially imported into EndNote reference management software to facilitate organization and systematic screening. Duplicate entries were identified and removed prior to further assessment. Subsequently, two independent reviewers screened the titles and abstracts of all unique articles using a set of predefined inclusion and exclusion criteria. Full-text versions of studies deemed potentially relevant were then obtained and evaluated for final eligibility. Studies were included if they fulfilled the following conditions: (1) examined the relationship between the SII and NAFLD; (2) reported relevant diagnostic or clinical outcomes pertaining to NAFLD; (3) provided sufficient data to compute effect estimates such as odds ratios (ORs), mean differences, or correlation coefficients; and (4) employed an observational study design, including cohort, case-control, or cross-sectional methodologies. Articles were excluded if they lacked adequate data for analysis or if they were in the form of conference abstracts, editorials, case reports, clinical trials, or narrative and systematic reviews. Any disagreements arising during the selection process were resolved through consensus between reviewers or with the involvement of a third reviewer when necessary.

### Quality assessment

The included studies were assessed for quality using the Joanna Briggs Institute risk of bias tool, evaluating domains such as design, conduct, and analysis^[31,32]^. Studies were rated as low risk (≥7/8 criteria met), medium risk (4–6/8), or high risk (<4/8). Any differences in judgment between reviewers were resolved through discussion to reach a consensus. Finally, the overall quality of the evidence was systematically summarized.

### Data extraction

Data were extracted using a standardized collection form. Extracted information included study details (first author, publication year, country, study design, and sample size), population demographics (age, gender, and comorbidities), specifics of SII assessment, NAFLD diagnostic methods, and main findings. In cases where numerical values were absent or only illustrated graphically, the study authors were contacted for clarification. If no response was received within 2 weeks, studies with insufficient extractable data were excluded. Data extraction was independently carried out by two reviewers, and any disagreements were resolved through discussion.

### Statistical analyses

Statistical analyses were carried out using STATA version 18 to integrate data from the selected studies. Standardized mean differences (Hedges’ *g*) were used by default for continuous SII analyses, and ORs for categorical data. To account for potential variability among studies, a random-effects model was applied to calculate pooled estimates. Heterogeneity among studies was evaluated using the *I*^2^ statistic, with values above 50% indicating significant heterogeneity. Sensitivity analyses were conducted by systematically excluding individual studies to assess their influence on the overall estimates. Additionally, prediction intervals were calculated to estimate the likely range of true estimates in future research, improving the interpretability of findings. Subgroup analyses were also performed to identify possible sources of heterogeneity.

## Results

### Study selection

**Figure 1.**
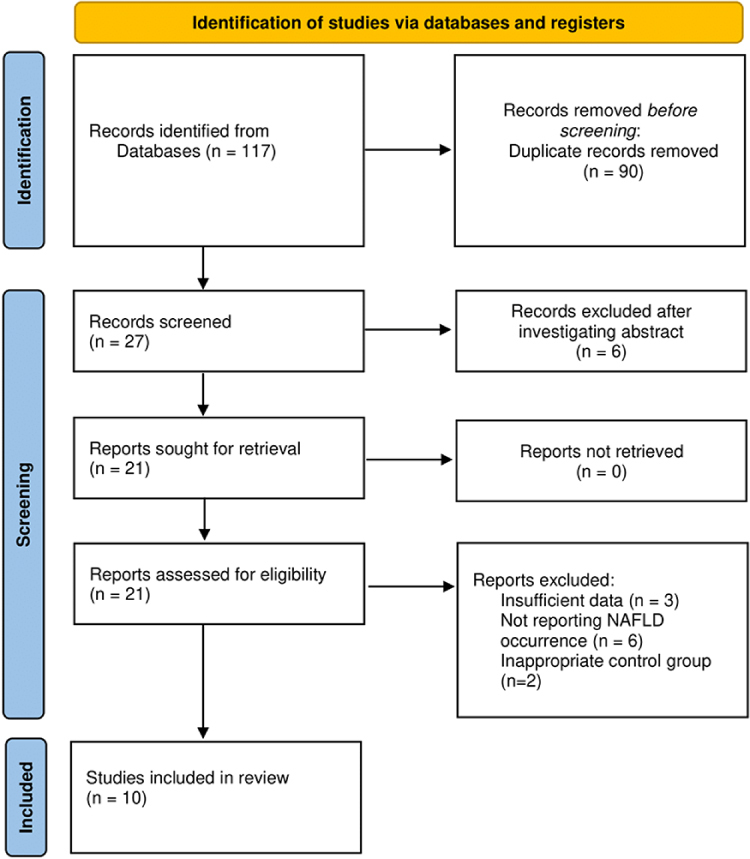
Study selection process.

A total of 117 records were initially retrieved through comprehensive database searches. Prior to the screening phase, 90 duplicate entries were identified and subsequently removed, resulting in 27 unique records eligible for preliminary screening. Titles and abstracts of these records were independently reviewed against the predefined eligibility criteria, leading to the exclusion of six studies. The remaining 21 articles were selected for full-text assessment. No reports were excluded due to retrieval issues at this stage. Following a thorough evaluation of the full texts, 11 studies were excluded for not meeting the inclusion criteria. Specifically, three studies lacked sufficient quantitative data for analysis, six did not report NAFLD-related outcomes, and two employed control groups that were deemed methodologically inappropriate. Consequently, a total of 10 studies satisfied all inclusion criteria and were incorporated into the final systematic review and meta-analysis. The full study selection process is illustrated in the PRISMA flow diagram (Fig. 1).

### Study characteristics

A total of ten (nine cross-sectional and one prospective cohort) studies published between 2022 and 2024 were included in the final synthesis, each examining the relationship between the SII and NAFLD. The majority of studies were conducted in China^[26,28,33–39]^, with one study originating from Turkey^[40]^. The temporal span of data collection ranged from 2006 to 2022, reflecting both historical and contemporary data sources. The included studies encompassed diverse populations, incorporating both pediatric^[40]^ and adult cohorts. Sample sizes varied substantially, ranging from small clinical samples to large-scale, population-based cohorts exceeding 378 000 individuals (e.g., Gong *et al*^[33]^).

There was notable heterogeneity in the demographic profiles of study participants. Pediatric data were reported in Tasolar *et al*, with mean ages of 11.6 ± 2.9 years for the control group and 12.4 ± 3.3 years for the NAFLD group^[40]^. In contrast, adult populations were represented in other studies, such as Wang *et al*, where mean ages were 62.47 ± 7.94 and 63.14 ± 7.41 years for the control and NAFLD groups, respectively^[36]^. Most studies reported gender distribution, which was generally balanced across comparison groups. Males ranged 45.1–55.8% across studies; two studies did not report the related data. As anticipated, mean BMI values were consistently elevated in individuals with NAFLD compared to healthy controls, with reported values ranging from 27.09 ± 3.25 to 34.33 ± 7.35 in NAFLD cohorts. Studies used the same BMI cut-offs, but most adjusted for BMI as a confounder in analyses; no uniform threshold was applied across studies. Nonetheless, some studies lacked comprehensive reporting of demographic or anthropometric variables. A detailed overview of study-level characteristics is presented in Table 1.

**Table 1:** Key characteristics of included studies

First author	Country	Year of publication	Time set	Study design	Study population (normal/NAFLD)	Mean age,^a^ years (normal/NAFLD)	Gender distribution (M/F)	BMI^a^ (normal/NAFLD)
Song *et al*^[34]^	China	2022	2015–2018	Cross-sectional	4568/5937	46.20 ± 0.60/48.77 ± 0.46	N.A.	23.79 ± 0.07/33.88 ± 0.15
Tasolar *et al*^[40]^	Turkey	2022	N.A.	Cross-sectional	70/63	11.6 ± 2.9/12.4 ± 3.3	60/73	27.4 ± 5.0/32.4 ± 13.7
Xie *et al*^[38]^	China	2022	2017–2020	Cross-sectional	3901/1031	46.135 ± 19.305/51.607 ± 17.552	N.A.	26.902 ± 5.946/31.480 ± 6.801
Gong *et al*^[33]^	China	2024	2006–2010	Cohort	216 230/161 909	56.40 ± 8.21/56.77 ± 7.91	203 049/175 090	24.48 ± 2.46/31.32 ± 4.23
Jiang *et al*^[26]^	China	2024	2017–2020	Cross-sectional	2817/2209	44.77 ± 18.44/50.78 ± 16.44	2408/2618	26.73 ± 5.71/34.33 ±7.35
Liu *et al*^[28]^	China	2024	2007–2018	Cross-sectional	7496/3325	N.A.	5299/5522	N.A.
Sun *et al*^[35]^	China	2024	2017–2018	Cross-sectional	863/336	44.02 ± 0.87/51.24 ± 1.60	585/614	28.72 ± 0.36/30.73 ± 0.77
Wang *et al*^[36]^	China	2024	June–December 2021	Cross-sectional	2382/2631	62.47 ± 7.94/63.14 ± 7.41	993/4020	24.98 ± 3.03/28.27 ± 3.36
Wang *et al*^[37]^	China	2024	2007–2018	Cross-sectional	7895/6518	48.16 ± 18.32/51.70 ± 16.28	7008/7405	N.A.
Zhao *et al*^[39]^	China	2024	January 2022–December 2022	Cross-sectional	25 494/11 599	N.A.	20 709/16 387	22.86 ±2 .84/27.09 ± 3.25

BMI, body mass index; F, Female; M, Male; N.A., not available; NAFLD, nonalcoholic fatty liver disease; SD, standard deviation.

^a^ Data presented as mean ± SD.

Furthermore, the overall methodological quality of the included studies was acceptable, with all studies exhibiting a low risk of bias. A detailed summary of the quality assessment is provided in Supplemental Digital Content Table S2, available at: http://links.lww.com/MS9/B2.

### Results of meta-analysis

#### SII as a continuous variable

**Figure 2.**
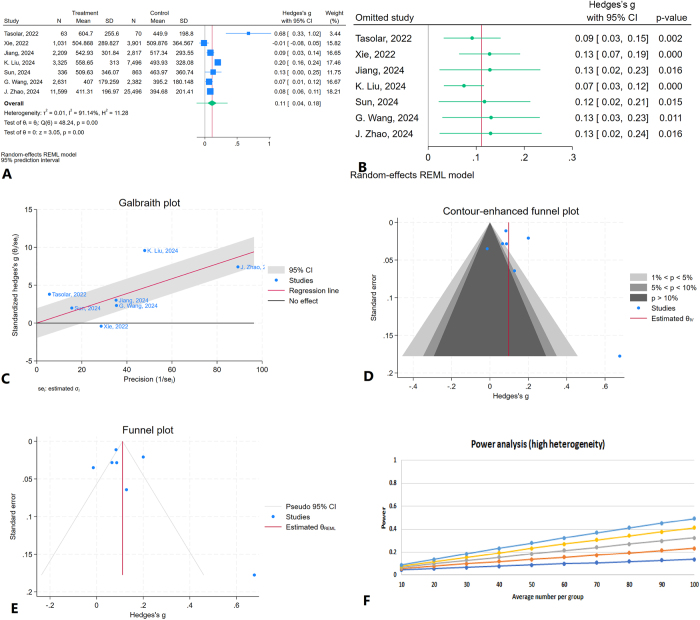
Meta-analysis of the association between the SII and NAFLD when SII is evaluated as a continuous variable. (A) Forest plot showing the pooled effect size (Hedges’ *g*) and 95% confidence intervals across the included studies. (B) Leave-one-out sensitivity analysis. (C) Galbraith plot. (D) Contour-enhanced funnel plot. (E) Funnel plot with the trim-and-fill method. (F) Power analysis result.

A total of seven studies were included in the meta-analysis evaluating the association between SII and NAFLD, with SII treated as a continuous variable. The pooled effect size indicated a statistically significant positive association between SII levels and NAFLD (Hedges’ *g* = 0.11, 95% CI: 0.04–0.18, *P* < 0.01) (Fig. 2A). Heterogeneity among the included studies was high, with an *I*^2^ value of 91.14% and a statistically significant *Q* test (*P* < 0.01), indicating substantial variability in effect sizes. A sensitivity analysis was conducted to assess the influence of each individual study on the overall results. The effect size remained statistically significant across all iterations, suggesting that no single study unduly influenced the findings with pooled Hedges’ *g* ranging from 0.09 to 0.13 (Fig. 2B).

To further explore heterogeneity, a Galbraith plot was generated (Fig. 2C). Most studies were within the 95% confidence interval range, although three studies^[28,38,40]^ appeared slightly divergent, potentially contributing to the observed heterogeneity. Assessment of publication bias using a contour-enhanced funnel plot suggested the possible presence of publication bias (Fig. 2D). Egger’s test supported this finding, showing statistically significant publication bias (*P* = 0.01), whereas Begg’s test did not indicate significant bias (*P* = 0.54). Furthermore, the trim-and-fill analysis did not impute any missing studies (Fig. 2E). Power analysis demonstrated high statistical power (exceeded 99%; alpha = 0.05), indicating that the meta-analysis had an adequate sample size to detect the observed effect (Fig. 2F).

#### SII as a categorical variable

**Figure 3.**
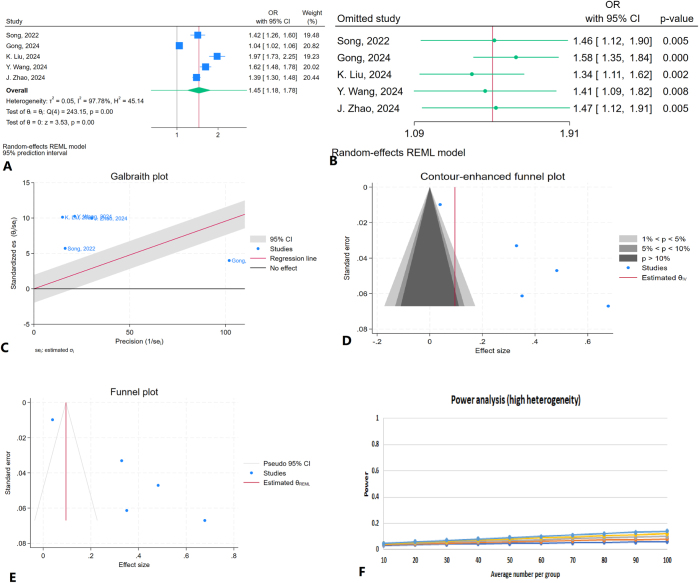
Meta-analysis of the association between the SII and NAFLD when SII is evaluated as a categorical variable. (A) Forest plot showing the pooled effect size (Hedges’ *g*) and 95% confidence intervals across the included studies. (B) Leave-one-out sensitivity analysis. (C) Galbraith plot. (D) Contour-enhanced funnel plot. (E) Funnel plot with the trim-and-fill method. (F) Power analysis result.

A total of five studies were included in the meta-analysis evaluating the association between SII and NAFLD, with SII treated as a categorical variable. The pooled analysis demonstrated a statistically significant positive association between elevated SII and the presence of NAFLD (OR = 1.45, 95% CI: 1.18–1.78, *P* < 0.01) (Fig. 3A). Heterogeneity among studies was considerable, with an *I*^2^ value of 97.78% and a significant *Q* test (*P* < 0.01). A leave-one-out sensitivity analysis showed that the pooled OR remained statistically significant regardless of which study was excluded (Fig. 3B).

A Galbraith plot was used to further explore heterogeneity (Fig. 3C). Remarkably, all included studies were located outside both the regression line and the 95% confidence interval bands, confirming a high degree of heterogeneity and suggesting a potential poor fit of the random-effects model due to inter-study variability. Publication bias was assessed using the funnel plot and Egger’s test. The funnel plot displayed an asymmetrical distribution of studies, suggesting potential publication bias (Fig. 3D). Egger’s test confirmed this with a statistically significant result (*P* < 0.01), whereas Begg’s test did not indicate significant bias (*P* = 0.27). The trim-and-fill analysis did not impute any missing studies (Fig. 3E), implying that, although bias is likely present, it may have limited influence on the pooled effect size. Publication bias was assessed using a contour-enhanced funnel plot. The funnel plot suggested potential publication bias (Fig. 3D). Egger’s test confirmed this with a statistically significant result (*P* < 0.01), whereas Begg’s test did not indicate significant bias (*P* = 0.27). The trim-and-fill analysis did not impute any missing studies (Fig. 3E). Power analysis revealed high statistical power (exceeded 99%; alpha = 0.05) (Fig. 3F).

## Discussion

This meta-analysis showed a significant association between elevated SII levels and NAFLD. Individuals with NAFLD had consistently higher SII values, both when analyzed as a continuous measure and when classified categorically. The association remained robust across sensitivity analyses and was supported by sufficient statistical power. Although considerable heterogeneity and some indication of publication bias were observed, the direction and consistency of findings suggest that SII serves as a potential marker for identifying individuals at risk of NAFLD.

The immune-inflammatory landscape of NAFLD is shaped by a complex interplay between innate and adaptive immune responses, oxidative stress, and metabolic dysfunction^[41–43]^. Within this context, elevated neutrophil and platelet counts along with decreased lymphocyte levels, which are key hematologic features captured by the SII, have been mechanistically linked to liver injury and fibrogenesis^[44–47]^. Neutrophils promote hepatocellular damage through the generation of reactive oxygen species (ROS), release of myeloperoxidase, and formation of neutrophil extracellular traps, contributing to oxidative stress, inflammation, and activation of hepatic stellate cells. Elevated ROS levels can cause extensive molecular damage to cellular membranes, proteins, and DNA, resulting in hepatocellular injury and apoptosis, facilitating progression from steatosis to NASH^[48–51]^. Platelets actively participate in hepatic inflammation by releasing pro-inflammatory mediators like platelet-derived growth factor, transforming growth factor-beta, and serotonin, which amplify inflammatory signaling, promote fibrotic remodeling, and enhance leukocyte infiltration^[52–55]^. In contrast, decreased lymphocyte counts, particularly of regulatory T cells (Tregs), may indicate impaired immune surveillance and a reduced capacity to counterbalance hepatic inflammation, allowing unchecked innate immune activation and contributing to sustained inflammation and progression toward steatohepatitis and fibrosis^[56–60]^.

Activation of lymphocytes and central granulocytes plays a pivotal role in initiating the release of pro-inflammatory cytokines, such as tumor necrosis factor-alpha, Interferon-gamma, and interleukins, including IL-1, IL-2, and IL-6. These inflammatory mediators disrupt the redox equilibrium within hepatocytes, thereby inducing oxidative stress and promoting ROS accumulation, which provokes further inflammatory responses, establishing a self-perpetuating cycle of oxidative injury and immune activation that ultimately contributes to the development and progression of NAFLD^[42,61–65]^. These cytokine-driven processes are captured by SII through shifts in neutrophil (elevated in response to cytokine signaling), lymphocyte (decreased due to suppression or apoptosis), and platelet counts (increased via activation and aggregation), providing a composite measure of the inflammatory imbalance.

Although the development of NAFLD is well-recognized to involve both metabolic and inflammatory processes, the conclusions of previous studies examining systemic immune-inflammatory biomarkers have been inconsistent. A number of earlier investigations reported weak or nonlinear associations between indices such as SII and NAFLD^[25,35,38,66]^. For instance, Zhao *et al* reported a nonlinear J-shaped association between log_2_-SII and all-cause mortality in NAFLD patients, with a threshold value of 8.8 log_2_-SII, suggesting that elevated SII levels may be prognostically relevant only beyond a certain threshold^[25]^. Similarly, Xie *et al* reported only a weak positive correlation between SII and hepatic steatosis, which was significant only in men, and found no significant association with liver fibrosis^[38]^. Moreover, Zhao *et al* found a U-shaped relationship between SII and NAFLD risk in a nationally representative U.S. cohort, with risk minimized at the SII index reached 422.40, indicating no consistent linear increase at higher levels^[66]^.

Despite these inconsistencies, the findings of this meta-analysis are in agreement with a growing body of observational research supporting a significant link between elevated SII levels and NAFLD. Several studies have demonstrated that patients with NAFLD exhibit higher SII values compared to metabolically healthy controls. For instance, Fu and Chen, in a large population-based study among 532 non-NAFLD and 133 NAFLD American adolescents, found that elevated SII was significantly associated with increased odds of NAFLD (OR = 3.505, 95% CI: 1.092–11.249, *P* < 0.05), indicating its relevance even in younger populations^[27]^. In addition, Liu *et al* evaluated multiple systemic inflammatory biomarkers in a cohort of 10 821 adults and reported that ln-transformed SII had the strongest independent association with NAFLD (OR=1.46, 95% CI: 1.27–1.69), even after adjusting for confounders such as age, sex, and metabolic parameters^[28]^.

Also, in a recent study by Xue *et al*, conducted among patients with chronic hepatitis B and coexisting NAFLD, elevated SII levels were found to be a significant independent predictor of cirrhosis progression. Patients in the high SII group had an 88.5% increased risk of developing cirrhosis (HR = 1.885), underscoring the prognostic value of SII in monitoring disease severity and progression in NAFLD-related liver pathology^[67]^. Although variation in effect size has been noted across studies, which is potentially due to differences in population characteristics, NAFLD definitions, and SII cut-off values, the directionality of the association remains consistent. Overall, these findings corroborate the results of the present meta-analysis and further support the role of SII as a reliable marker of systemic inflammation in NAFLD.

Notably, in some studies, SII has demonstrated superior predictive value for NAFLD compared to other commonly utilized inflammatory markers. As highlighted in a previously discussed study by Liu *et al*, SII exhibited a stronger and more consistent association with NAFLD risk than other systemic biomarkers. The OR for SII was 1.52, which was higher than that of the neutrophil-to-lymphocyte ratio (OR = 1.31), platelet-to-lymphocyte ratio (OR = 1.18), and lymphocyte-to-monocyte ratio (OR = 0.82)^[28]^. These findings suggest that, by incorporating multiple components of the innate and adaptive immune response, SII may provide a more comprehensive reflection of the complex immunopathogenesis underlying NAFLD.

The consistent association between elevated SII values and the presence of NAFLD, as demonstrated in this meta-analysis, underscores the potential of SII as a clinically meaningful biomarker for early risk stratification. As an index derived from routinely obtained hematologic parameters, including neutrophils, lymphocytes, and platelets, SII offers several practical advantages, including accessibility, low cost, and ease of integration into standard laboratory workflows. Although the head-to-head comparisons are lacking, compared to established noninvasive indices such as the NAFLD fibrosis score or FIB-4, which require multiple biochemical and clinical inputs, SII reflects the systemic inflammatory milieu that underpins NAFLD pathogenesis, potentially offering complementary prognostic information. Its incorporation into routine clinical evaluation may facilitate the identification of at-risk individuals, particularly in primary care or low-resource settings. However, shortcomings must be considered, including variability across hematology analyzers, transient neutrophil elevations due to infections, and influences from medications like steroids or antiplatelet agents, which could affect reliability^[33,68–70]^. Despite these, incorporating SII into routine evaluations may serve as a candidate marker for longitudinal monitoring of disease activity or response to therapeutic interventions.

### Limitations and future directions

This study presents several limitations that should be considered when interpreting the findings. First, the presence of high heterogeneity across included studies may influence the precision of the pooled estimates. The number of studies included in the analysis was relatively small without sufficient data, which limited our ability to perform subgroup analyses and meta-regression to identify potential sources of heterogeneity. Specifically, data for the stage of NAFLD patients were not consistently reported across studies, and there was insufficient data to separately analyze SII levels in male and female participants. Additionally, due to the low number of effect sizes (fewer than 10), meta-regression analysis was not feasible. Variability in study design, population characteristics, diagnostic criteria for NAFLD, and cut-off values for SII likely contributed to heterogeneity. Additionally, variations in SII calculation, such as different complete blood count technologies, and different timing of blood draws, may introduce bias and affect comparability. Third, although publication bias was assessed and adjusted for, the potential underreporting of negative or nonsignificant results cannot be entirely ruled out. Moreover, the reliance on observational studies limits the ability to establish causality between elevated SII and NAFLD. Lastly, some included studies lacked comprehensive reporting of confounding variables, which may have affected the strength of observed associations.

Furthermore, future studies should evaluate SII with new terminology such as MASLD and other emerging terms, as using different terminology could affect the comparability of findings and the interpretation of results in future research. Future research should also focus on prospective, large-scale studies with standardized definitions of SII and NAFLD to improve comparability across populations. Additionally, mechanistic studies are needed to clarify the biological pathways linking systemic inflammation and liver disease progression. Incorporating SII into clinical risk models and comparing its predictive power against established biomarkers may also help determine its practical utility in NAFLD diagnosis and monitoring.

## Conclusion

This systematic review and meta-analysis highlight a significant association between the SII and NAFLD. Elevated SII levels were consistently linked to increased NAFLD risk, underscoring the index’s potential as a simple, cost-effective, and noninvasive biomarker. While the findings are promising, methodological limitations and inter-study variability necessitate cautious interpretation. Further validation through well-designed prospective studies is essential, with optimal follow-up ≥5 years to assess SII’s predictive value for fibrosis progression, and standardization of confounder adjustments (diabetes status and lipid medications) to confirm the clinical relevance of SII and to explore its integration into routine NAFLD screening and management strategies.

## Data Availability

The datasets used and/or analyzed during the current study can be provided by the corresponding author on reasonable request.

## Research registration unique identifying number (UIN)

The study protocol was registered in PROSPERO with the registration code CRD42024578894.
